# Adjuvant commensal therapy enhances antibiotic efficacy against *Staphylococcus aureus*

**DOI:** 10.1186/s40168-026-02479-6

**Published:** 2026-08-14

**Authors:** Chun-Chi Chang, Tomas Demeter, Alejandro Gómez-Mejia, Weronika Barcik, Judith Bergada-Pijuan, Andrew G. Kempchinsky, Umberto Maccio, Eline Cauwenberghs, Nora Ranzoni, Ilke De Boeck, Sarah Lebeer, Srikanth Mairpady Shambat, Silvio D. Brugger

**Affiliations:** 1 Department of Infectious Diseases and Hospital Epidemiology, University Hospital Zurich, University of Zurich, Zurich, Switzerland; 2 Institute of Pathology and Molecular Pathology, University Hospital Zurich, University of Zurich, Zurich, Switzerland; 3 Department of Bioscience Engineering, University of Antwerp, Antwerp, Belgium

**Keywords:** *Staphylococcus aureus*, *Dolosigranulum pigrum*, *Corynebacterium* spp., Nasal microbiota, Antibiotic adjuvant, 3D humanized airway tissue model, Dual RNA-seq

## Abstract

**Background:**

*Staphylococcus aureus* is a common human pathobiont whose nasal colonization represents a major risk factor for subsequent infection. The prevalence of antimicrobial resistance, coupled with antibiotic-induced microbiota perturbation, has rendered *S. aureus*-associated infections increasingly difficult to treat. Commensal bacteria have emerged as potential modulators of both host immune responses and pathogen pathogenesis; however, whether they can act as therapeutic adjuvants to enhance treatment outcomes in *S. aureus-*associated infections remains unclear. Here, we assess the efficacy of commensal bacteria as an adjuvant therapeutic strategy.

**Results:**

Using A549 Dual™ reporter cells, we demonstrate that commensals and pathobionts elicit distinct activation profiles of NF-κB and interferon regulatory factor (IRF) signaling pathways and differential IL-8 production. In a 3D humanized airway tissue model, *D. pigrum* significantly enhanced antibiotic-mediated reduction of *S. aureus* survival relative to antibiotic treatment alone (*D. pigrum* KPL1914: 37.3% reduction, 95% CI 2.0–72.6%, *P*_adj_ = 0.0340; *D. pigrum* AMBR11: 48.1% reduction, 95% CI 2.3–93.9%, *P*_adj_ = 0.0356), while preserving barrier integrity and augmenting pro-inflammatory cytokine responses. However, the loss of efficacy with heat-killed *D. pigrum* indicates that viable bacteria and active host- and/or pathobiont-directed interactions are required to mediate its anti-*S. aureus* effects. Dual RNA-seq transcriptomic analysis revealed enrichment of host inflammatory pathways and reduction of *S. aureus* pathogenic potential during *D. pigrum* antibiotic adjuvant treatment in the airway tissue model. Furthermore, co-culture experiments revealed that *D. pigrum* constrains core metabolic pathways in *S. aureus*.

**Conclusions:**

These findings demonstrate that *D. pigrum* limits *S. aureus* through both host immune modulation and direct suppression of bacterial pathogenesis and adaptive fitness, supporting its potential as a microbiota-based therapeutic adjuvant to enhance antibiotic efficacy against *S. aureus*-associated infections.

Video Abstract

**Supplementary Information:**

The online version contains supplementary material available at https://doi.org/10.1186/s40168-026-02479-6.

## Introduction

*Staphylococcus aureus* is an important human pathobiont that colonizes approximately one-third of the human population and constitutes an important reservoir for subsequent infection [1–3]. Colonization by *S. aureus* plays a pivotal role in the pathogenesis of chronic rhinosinusitis (CRS) and it is a frequent cause of skin and soft tissue infections (SSTIs) [4–11]. Beyond these localized conditions, *S. aureus* colonization has also been associated with invasive infections including pneumonia and bacteremia, as well as autoimmune disorders such as granulomatosis with polyangiitis [12–17]. These clinical manifestations are often characterized by recurrent exacerbations, prolonged treatment courses, and a substantial healthcare burden [18–21].

The treatment of *S. aureus*-associated infections has traditionally relied on antibiotic and antiseptic therapy [18, 22–25]. However, the growing prevalence of antibiotic resistance within *S. aureus* populations, driven by selective pressure exerted by antibiotics, and the remarkable adaptability of *S. aureus* to evade action of antimicrobial agents has profoundly compromised the efficacy of conventional treatment regimens [26–31]. Furthermore, antibiotic and antiseptic-perturbed (dysbiotic) microbiota have been linked to imbalanced metabolism and dysregulated innate immunity of the human host, adding layers of complexity in pathogenesis and management [32–37]. Given these challenges, there is a compelling need to explore alternative therapeutic strategies that circumvent antibiotic resistance mechanisms, restore microbiota homeostasis, and modulate host immune responses to tackle *S. aureus*-associated chronic inflammation and infection effectively [38].

Prior 16S rRNA gene amplicon sequencing studies have demonstrated an increased abundance of the nasal bacterium *Dolosigranulum pigrum* in individuals who do not carry *S. aureus*. Similarly, *S. aureus* colonization was associated with reduced or absent *D. pigrum,* suggesting an inverse relationship between the two species [39–42]. While *D. pigrum* was initially described in a disease-associated context in 1993 [43], subsequent studies began to recognize and appreciate its commensal nature with mutualistic associations with human health and inverse relationships with infectious diseases [44–49]. In line with this, *D. pigrum* lacks predicted virulence factors and exhibits direct inhibitory activity against *S. aureus* [40, 50, 51], positioning it as both a predictor of *S. aureus* exclusion and a promising candidate for microbiota-targeted respiratory therapies against respiratory tract infections [52–54]. Recent work has further shown that *D. pigrum* can colonize human nasal epithelial organoids and modulate the production of multiple cytokines and chemokines [55]. Moreover, *S. aureus*-induced pro-inflammatory cytokine response was attenuated when co-cultured with nasal strain *D. pigrum* AMBR11 in human airway epithelial cells [51], highlighting the capacity of *D. pigrum* to modulate host responses through host-bacteria interactions. However, the molecular mechanisms by which *D. pigrum* modulates host immune responses and influences *S. aureus* pathogenicity remain unclear. In addition, its potential as a live biotherapeutic product (LBP) to enhance antibiotic efficacy against *S. aureus* in a host-like environment has yet to be determined. The aim of this study was therefore to investigate the efficacy of *D. pigrum* as an adjuvant LBP against *S. aureus* respiratory tract infections.

## Results

### Differential activation of innate immune signaling pathways by nasal commensals and pathobionts

**Fig. 1:**
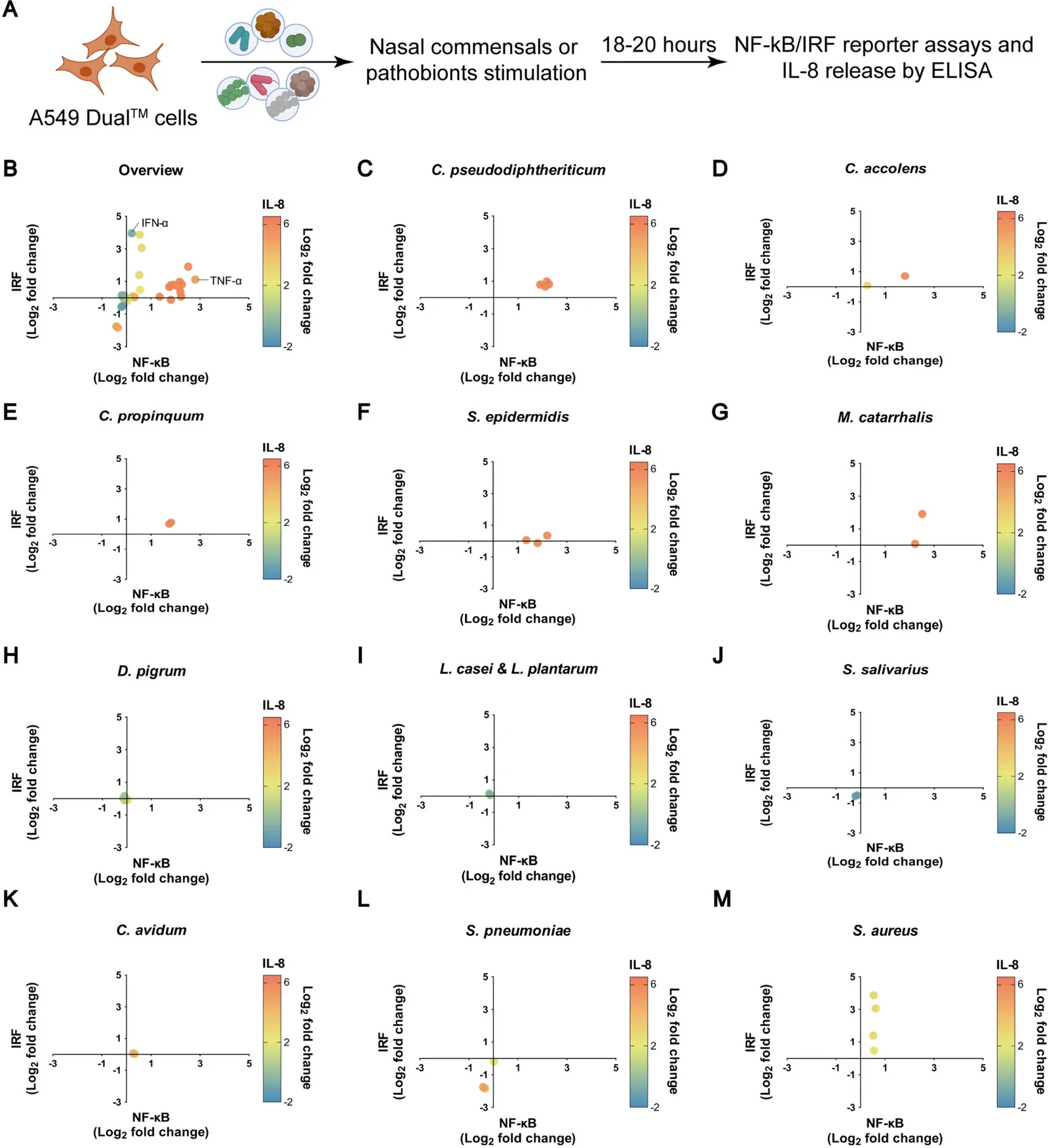
Activation of innate signal transduction by nasal commensals and pathobionts in A549 Dual™ cells. **A** Schematic of experimental setup of measuring NF-κB/IRF induction and IL-8 release in A549 Dual™ cell. A549 Dual™ cells were stimulated with commensals or pathobionts (Table 1) at MOI 1 for 18–20 h. Induction of NF-κB and IRF were determined by the activity of SEAP and Lucia luciferase in the supernatant as described in the “Methods” section. IL-8 secretion in the supernatant was determined by ELISA. *S.* *aureus* and *S.* *pneumoniae* stimulating wells were added with 4× MIC of flucloxacillin (+ Abx) 3 h post-stimulation. **B**–**M** Multivariate display of NF-κB, IRF and IL-8 induced by commensals and pathobionts for 18–20 h in A549 Dual™ cells. Dot color indicates IL-8 levels. All values (NF-κB, IRF, and IL-8) were log_2_-transformed and expressed as fold change relative to the negative control (medium only). Each dot represents the mean of four to six biological replicates for a given bacterial strain

To deepen our understanding of immune signal transduction in response to various nasal commensals and pathobionts, we evaluated a total of 37 bacterial isolates, from seven genera and twelve species. The majority of isolates were derived from the upper respiratory tract of healthy individuals, except for several pathobiont reference strains, including *S. aureus* (Cowan I, JE2), *S. pneumoniae* (D39, TIGR4), and *Moraxella catarrhalis* isolates obtained from clinical specimens (Table 1). We assessed their ability to activate the global transcriptional regulators NF-κB and IRFs using dual reporter assays, as well as their induction of cytokine IL-8, a potent signal for immune cell recruitment and activation at barrier tissues [60–62], in the supernatants of A549 Dual™ cells (Fig. 1 and Supplementary Fig. S1A–C). Cytokine TNF-α and IFN-α served as positive controls for NF-κB and IRF induction, respectively (Fig. 1A). Minimal cell cytotoxicity was observed in cells stimulated with the tested bacterial species (Supplementary Fig. S1D). Our findings show that *Corynebacterium pseudodiphtheriticum*, *Corynebacterium accolens*, *Corynebacterium propinquum*, *Staphylococcus epidermidis*, and *M. catarrhalis* primarily activated the NF-κB pathway, showing a one to two-fold increase compared to the medium control (Fig. 1C–G, Supplementary Fig. S1A). In contrast, *D. pigrum, Lacticaseibacillus casei*, *Lactiplantibacillus plantarum, Streptococcus salivarius*,* Cutibacterium avidum*, and* S. pneumoniae* did not activate the NF-κB or IRF pathways in A549 Dual™ cells. (Fig. 1H–L, Supplementary Fig. S1A–1B). Notably, *S. aureus* (in three out of four isolates tested) activated IRFs with minimal activation of NF-κB pathway (Fig. 1M, Supplementary Fig. S1A–1B). Additionally, our findings of the pro-inflammatory cytokine IL-8 release revealed that *D. pigrum* (across all eleven isolates tested), *Lactobacillus* spp.*,* and *S. salivarius* induced a minimal increase in IL-8 production (Fig. 1H–J, Supplementary Fig. S1C). This contrasted with the elevated IL-8 secretion observed with other commensals, including *Corynebacterium* spp., *S. epidermidis*, and *C. avidum* as well as pathobionts, such as *M. catarrhalis*, *S. pneumoniae*, and *S. aureus* (Fig. 1, Supplementary Fig. S1C). Importantly, the lack of NF-κB and IRF activation and the minimal IL-8 secretion in response to *D. pigrum, Lactobacillus* spp., and *S. salivarius*, was not due to cytotoxic effects, as assessed by the measurement of host-derived lactate dehydrogenase (LDH) release (Supplementary Fig. S1D). Together, these results demonstrate species-specific activation of innate immune signaling pathways and cytokine release in epithelial cells.

**Table 1:** List of bacterial isolates used in A549 Dual™ cell reporter assays

Isolate	Source	Species	Reference
C21	Nasal swab*	*S. aureus*	This study
46443	Nasal swab*	*S. aureus*	This study
Cowan I	Reference strain	*S. aureus*	MSSA, ST30, ATCC 12598 [56]
JE2	Reference strain	*S. aureus*	CA-MRSA, USA300, ST8, NARSA NR-46543
C6	Nasal swab*	*S. epidermidis*	This study
C5	Nasal swab*	*S. epidermidis*	This study
C33	Nasal swab*	*S. epidermidis*	This study
603	Nasal swab*	*S. pneumoniae*	Serotype 6B [40]
D39	Reference strain	*S. pneumoniae*	Serotype 2 NCTC 7466
TIGR4	Reference strain	*S. pneumoniae*	Serotype 4 ATCC BAA-334 [57]
3667	Clinical isolate*	*M. catarrhalis*	This study
8381	Clinical isolate*	*M. catarrhalis*	This study
KPL1989	Nasal swab*	*C. pseudodiphtheriticum*	[40]
CCC2	Nasal swab*	*C. pseudodiphtheriticum*	This study
Cps24	Nasal swab*	*C. pseudodiphtheriticum*	This study
C20	Nasal swab*	*C. pseudodiphtheriticum*	This study
C4	Nasal swab*	*C. accolens*	This study
C23	Nasal swab*	*C. accolens*	This study
C9	Nasal swab*	*C. propinquum*	This study
C13	Nasal swab*	*C. propinquum*	This study
C14	Nasal swab*	*C. avidum*	This study
C16	Nasal swab*	*C. avidum*	This study
KPL1914	Nasal swab*	*D. pigrum*	[40]
KPL1931	Nasal swab*	*D. pigrum*	[40]
KPL1939	Nasal swab*	*D. pigrum*	[40]
D5023	Nasal swab*	*D. pigrum*	This study
CCC1	Nasal swab*	*D. pigrum*	This study
CCC4	Nasal swab*	*D. pigrum*	This study
C24	Nasal swab*	*D. pigrum*	This study
C32	Nasal swab*	*D. pigrum*	This study
C40	Nasal swab*	*D. pigrum*	This study
C51	Nasal swab*	*D. pigrum*	This study
AMBR2	Nasal swab*	*L. casei*	[58]
AMBR11	Nasal swab*	*D. pigrum*	[51]
AMBR24	Nasal swab*	*S. salivarius*	[59]
AMBR24-1	Nasal swab*	*L. plantarum*	Gift from Sarah Lebeer lab
AMBR158	Nasal swab*	*S. salivarius*	[59]

^*^Identified by MALDI-TOF; *NARSA* network of antimicrobial resistance in *S. aureus*

### Treatment with *D. pigrum* as an antibiotic adjuvant reduces *S. aureus* survival in the airway tissue model

**Fig. 2:**
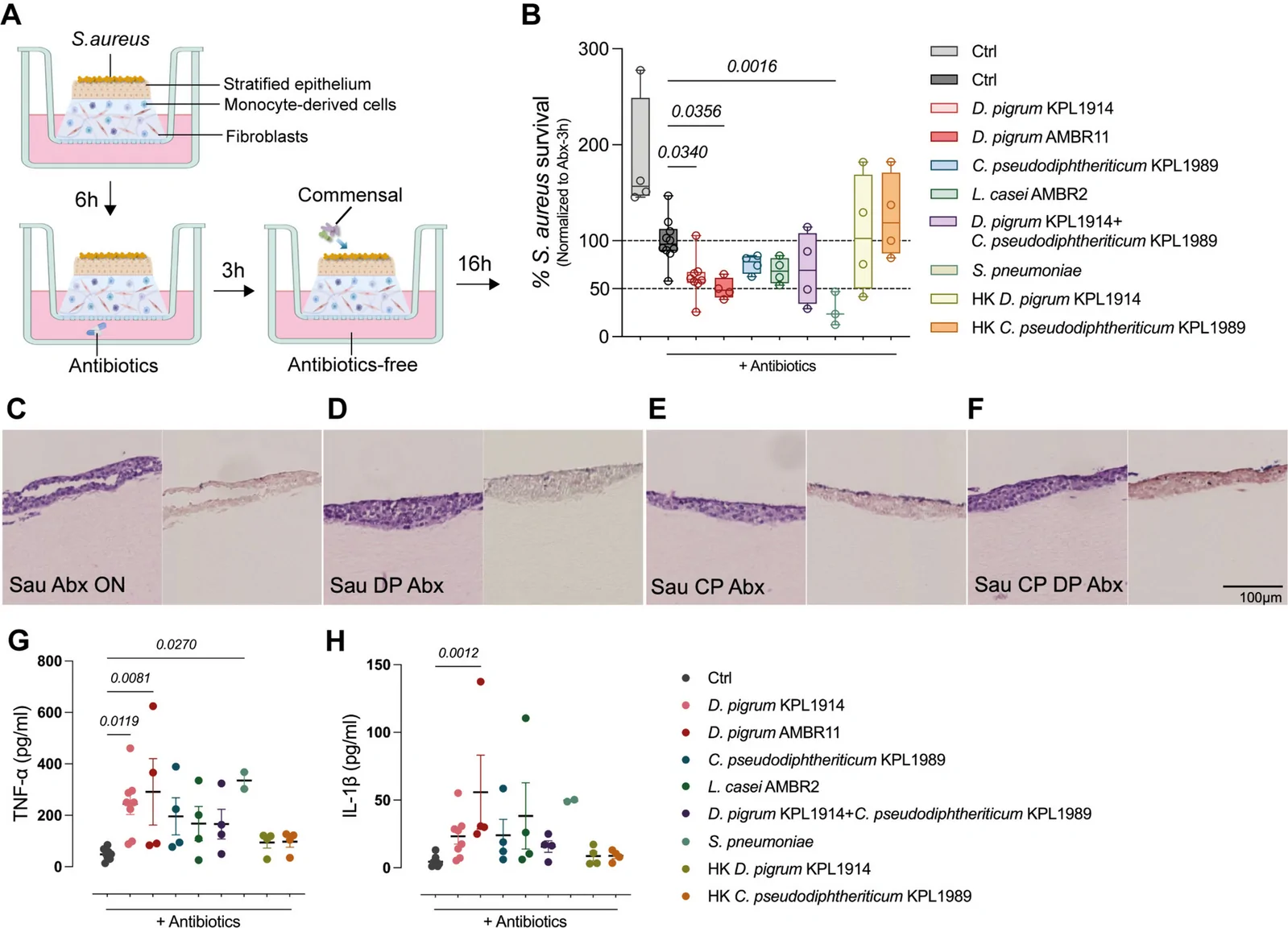
Nasal commensal as antibiotic adjuvant therapy reduces *S.* *aureus* survival and enhances cytokine response in the airway tissue model. **A** Schematic of experimental setup of antibiotic adjuvant therapy in the airway tissue model. **B** *S. aureus* survival in the airway tissue model in the presence of live or heat-killed (HK) commensal bacteria as antibiotic adjuvant therapy. Percentage was calculated by bacterial CFUs normalized to the end of 3 h antibiotic treatment time point. *S. aureus* alone, with or without antibiotics, served as the control (Ctrl) (data represent mean ± SEM. *n* = 3–10). Relative to antibiotics alone control, *D.* *pigrum* KPL1914 and AMBR11 reduced *S. aureus* survival by 37.3% (95% CI 2.0–72.6%; adjusted *p* = 0.0340) and 48.1% (95% CI 2.3–93.9%; adjusted *p* = 0.0356), respectively. **C**–**F** Representative hematoxylin and eosin (H&E)-stained histological sections of airway tissue model infected with *S. aureus* and treated with antibiotic alone (**C**), *D*. *pigrum* KPL1914 (**D**), *C*. *pseudodiphtheriticum* (**E**), or a combination of *D.* *pigrum* KPL1914 and *C.* *pseudodiphtheriticum* (**F**). **G**–**H** Culture supernatants were collected after 16 h with or without the addition of commensal bacteria, and TNF-α (**G**) and IL-1β (**H**) levels were quantified by ELISA (Data represent mean ± SEM. *n* = 2–8). Relative to antibiotics alone control, TNF-α increased 5.1-fold (adjusted *p* = 0.0119) and 6.2-fold (adjusted *p* = 0.0081) with *D.* *pigrum* KPL1914 and AMBR11, respectively, and IL-1β increased 13.0-fold with *D.* *pigrum* AMBR11 (adjusted *p* = 0.0012). Statistical significance was determined by one-way ANOVA with Dunnett’s multiple comparisons test; adjusted *p* values are indicated (**B**, **G**, and **H**)

To assess whether commensal bacteria can serve as an antibiotic adjuvant treatment against pathobiont infection, several commensal candidates exhibiting minimal activation of host innate immune signaling pathways, including *D. pigrum* and *L. casei*, were selected for exploring their adjuvant therapeutic effects against *S. aureus* in a 3D humanized airway tissue model (Fig. 2A). In addition, because *D. pigrum* and *C. pseudodiphtheriticum* frequently co-occur in the healthy nasal microbiome [39, 40, 42], *C. pseudodiphtheriticum* was also included to assess its potential adjuvant effects against *S. aureus* in the airway tissue model. This model includes a stromal matrix embedded with fibroblasts and primary monocyte-derived cells covered with stratified air-exposed epithelium on top (Fig. 2A) [63–65]. The model was infected with *S. aureus*, followed by the addition of flucloxacillin to the basal compartment for 3 h, mimicking systemic antibiotic administration in a clinical setting. Subsequently, commensal bacteria were inoculated to the apical surface and incubated overnight, while the basal compartment was maintained in antibiotic-free medium (Fig. 2A). The results revealed that commensal *D. pigrum* strains KPL1914 and AMBR11 significantly reduced the survival of *S. aureus* in the model (59.6% and 48.9% survival, respectively), corresponding to a 37.3% reduction (95% CI 2.0–72.6%; *P*_adj_ = 0.0340; *n* = 8) and a 48.1% reduction (95% CI 2.3–93.9%; *P*_adj_ = 0.0356; *n* = 4) relative to the antibiotics-alone control (97.0% survival, *n* = 10) (Fig. 2B). This antibacterial effect appeared to require active interspecies and host-bacteria interactions, as the use of heat-killed *D. pigrum* strain KPL1914 with antibiotics did not sustain this therapeutic effect against *S. aureus* (Fig. 2B). The conjunction of antibiotics with other commensals, such as *L. casei* and *C. pseudodiphtheriticum*, exhibited a similar trend but did not significantly reduce *S. aureus* survival (Fig. 2B). Likewise, heat-killed *C. pseudodiphtheriticum* alone showed no improvement of antibiotic efficacy against *S. aureus* (Fig. 2B). While *D. pigrum* and *C. pseudodiphtheriticum* frequently co-occur in the human nasal microbiome [39, 40, 42], the anti-*S. aureus* therapeutic was not enhanced when both were present as antibiotic adjuvant treatment (live *D. pigrum* KPL1914 together with live *C. pseudodiphtheriticum* KPL1989) compared with *D. pigrum* alone (Fig. 2B). *S. pneumoniae*, a pathogenic lactic acid bacterium also reduced *S. aureus* survival in the tissue model (Fig. 2B). However, this was likely due to bacterial invasion and *S. pneumoniae* overgrowth in the model (Supplementary Fig. S2A). In contrast, colonization by *D. pigrum* strain KPL1914 or *C.* *pseudodiphtheriticum* alone did not result in overgrowth in the model (Supplementary Fig. S2A). It is worth noting that L-lactate production, one of the mechanisms exerted by lactic acid bacteria for immunomodulatory effects, remained at similar levels as compared to control in *D. pigrum* and *L. casei* antibiotic adjuvant treatments (Supplementary Fig. S2B). Infection with *S. aureus* alone, or followed by antibiotic treatment, disrupted epithelial architecture and reduced tissue continuity, as demonstrated by histological staining (Fig. 2C and Supplementary Fig. S3). In contrast, colonization with *D. pigrum* KPL1914 or *C. pseudodiphtheriticum* KPL1989 preserved barrier integrity (Fig. 2D–F and Supplementary Fig. S3). This could be observed both during colonization alone and when used as antibiotic adjuvants (Fig. 2D–F and Supplementary Fig. S3). Together, our findings suggest that specific nasal commensals can serve as antibiotic adjuvant therapy against *S. aureus*.

To further investigate how antibiotic adjuvant therapy influences the host’s inflammatory response, we analyzed the basolateral supernatants from the airway tissue model to determine cytokine productions, namely TNF-α and IL-1β (Fig. 2G, H). The results showed that *D. pigrum* antibiotic adjuvant therapies, which improved antibiotic efficacy against *S. aureus* by decreasing *S. aureus* survival, were associated with increased cytokine production, including a 5.1-fold and 6.2-fold increase in TNF-α induced by the KPL1914 and AMBR11 strains (*Padj* = 0.0119, *n* = 8; and *Padj* = 0.0081, *n* = 4; respectively), as well as a 13.0-fold increase in IL-1β induced by the AMBR11 strain (*P*_adj_ = 0.0012, *n* = 4) (Fig. 2G, H). Consistent with previous findings, heat-killed *D. pigrum* with antibiotics, which did not improve antibiotic efficacy against *S. aureus*, was also unable to augment TNF-α and IL-1β production (Fig. 2G, H). This suggests that a more robust activation of the host’s innate immunity correlates with higher effectiveness of antibiotic treatments in the presence of commensal bacteria in the airway tissue model.

### *D. pigrum* antibiotic adjuvant treatment enhances immune inflammatory pathways against *S. aureus* in the airway tissue model

**Fig. 3:**
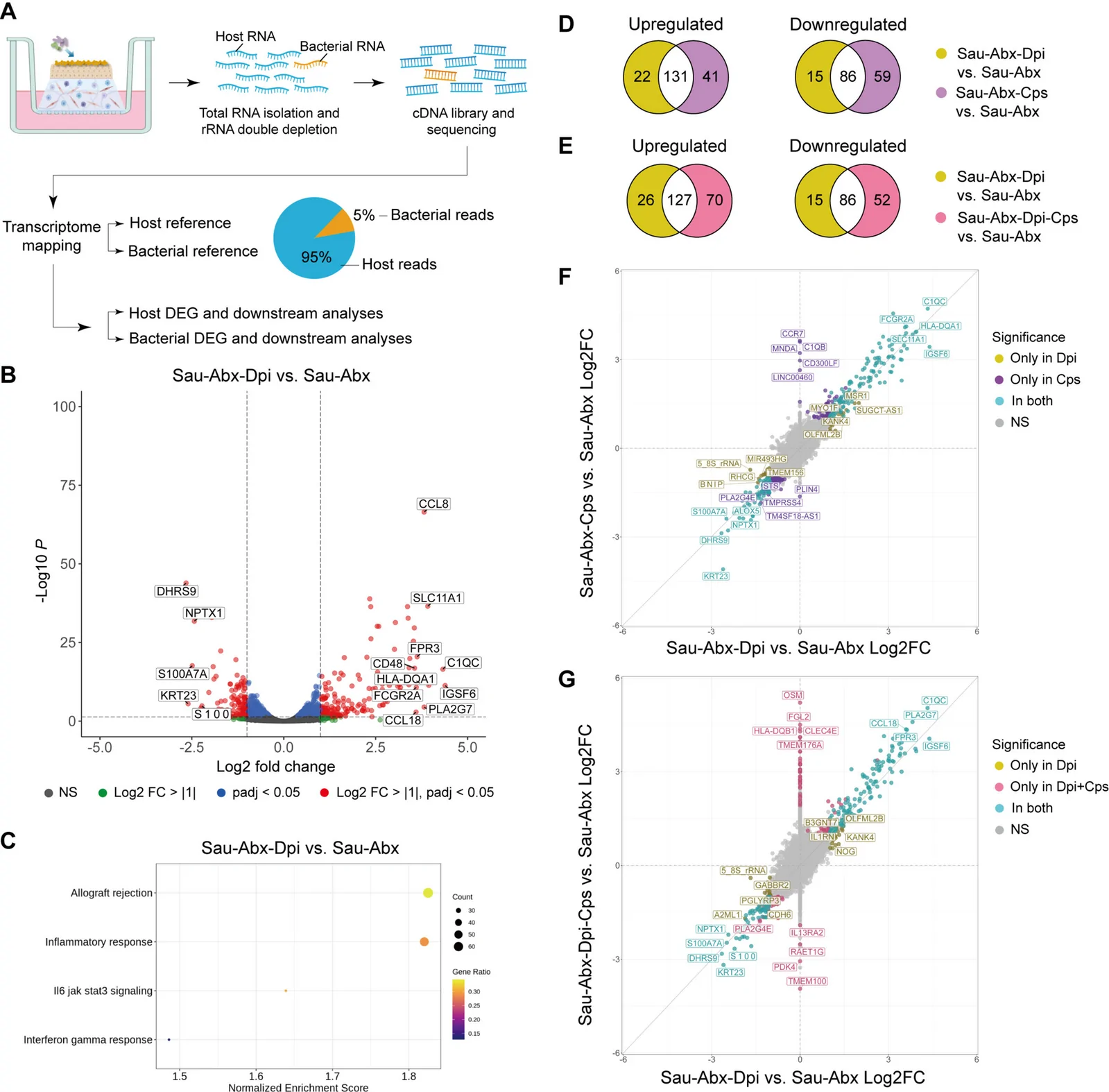
*D. pigrum* augments host inflammatory transcriptional responses to antibiotic treatment against *S.* *aureus* in the airway tissue model. **A** Schematic of experimental setup for dual RNA-seq analysis in the airway tissue model. **B** Volcano plot depicting differentially expressed host genes regulated by *D.* *pigrum* (Dpi) as an antibiotic adjuvant compared with antibiotic treatment (Abx) alone against *S.* *aureus* (Sau). The top 10 upregulated and top 5 downregulated genes are shown. **C** Enriched MSigDB Hallmark pathways regulated by *D.* *pigrum* as antibiotic adjuvant against *S.* *aureus*. **D**, **E** Venn diagrams summarizing host unique and overlapping differentially expressed genes (DEGs) with fold change |FC|≥ 1. **D** Comparison of host unique and shared DEGs between *D.* *pigrum* and *C*. *pseudodiphtheriticum* (Cps) antibiotic adjuvant treatments against *S. aureus*. **E** Comparison of host unique and shared DEGs between *D.* *pigrum* and combined *D.* *pigrum* + C*. pseudodiphtheriticum* (Dpi-Cps) antibiotic adjuvant treatments against *S. aureus*. **F**–**G** Scatter plots comparing log₂ fold changes (Log2FC) in host transcript abundance between commensal adjuvant treatments and antibiotic treatment alone. **F** The *x*-axis represents *D.* *pigrum* and the *y*-axis represents *C*. *pseudodiphtheriticum* treatments, both in the context of *S. aureus* infection. **G** The *x*-axis represents *D*. *pigrum* and the *y*-axis represents combined *D.* *pigrum* + *C.* *pseudodiphtheriticum* treatment, both in the context of *S. aureus* infection. Blue dots indicate commonly regulated transcripts (|FC|≥ 1), whereas yellow and purple dots represent DEGs unique to commensal-specific antibiotic adjuvant treatments (*n* = 2)

To further explore how antibiotics combined with adjuvant commensal treatment shapes host immune response against *S. aureus* and whether the commensal itself directly influences *S. aureus* pathogenicity, we performed dual RNA sequencing to profile both host and bacterial transcriptomic changes in the airway tissue model (Fig. 3A). Dual RNA-seq transcriptomic analysis identified 153 upregulated and 101 downregulated differentially expressed genes (DEGs) of host transcripts in the *D. pigrum* KPL1914 treatment group compared with antibiotic treatment alone (Fig. 3B). Among these host DEGs, we observed upregulation of chemotactic and trafficking molecules (e.g., *CCL8, CCL18, FPR3*), antigen presentation and costimulatory molecules (e.g., *HLA-DQA1, CD48, IGSF6*), as well as components of complement and phagocytosis pathways (e.g., *C1QC, SLC11A1, FCGR2A*) (Fig. 3B). In contrast, host genes involved in inflammation (e.g., *S100A7A, S100P*) and lipid metabolism (e.g., *DHRS9*) were downregulated (Fig. 3B). Gene set enrichment analysis (GSEA) further revealed significant enrichment of MSigDB Hallmark pathways associated with allograft rejection, inflammatory response, IL-6-JAK-STAT3 signaling, and interferon-γ response (Fig. 3C and Supplementary Fig. S4). Notably, within the allograft rejection pathway, genes involved in antigen presentation, including CD80, CD86, and HLA-DQA1, were significantly upregulated, suggesting enhanced antigen-presenting capacity of monocyte-derived macrophages in the tissue model (Supplementary Fig. S4). Although combined antibiotic adjuvant treatment with live *D. pigrum* KPL1914 and live *C. pseudodiphtheriticum* did not further reduce *S. aureus* survival (Fig. 2B), *C. pseudodiphtheriticum* alone or in combination with *D. pigrum* KPL1914 was associated with enrichment of MSigDB Hallmark pathways related to allograft rejection and inflammatory responses (Supplementary Fig. S5). Furthermore, *C. pseudodiphtheriticum* in combination with antibiotic treatment enriched pathways associated with KRAS signaling up and TNF-α signaling via NF-κB (Supplementary Fig. S5B).

**Fig. 4:**
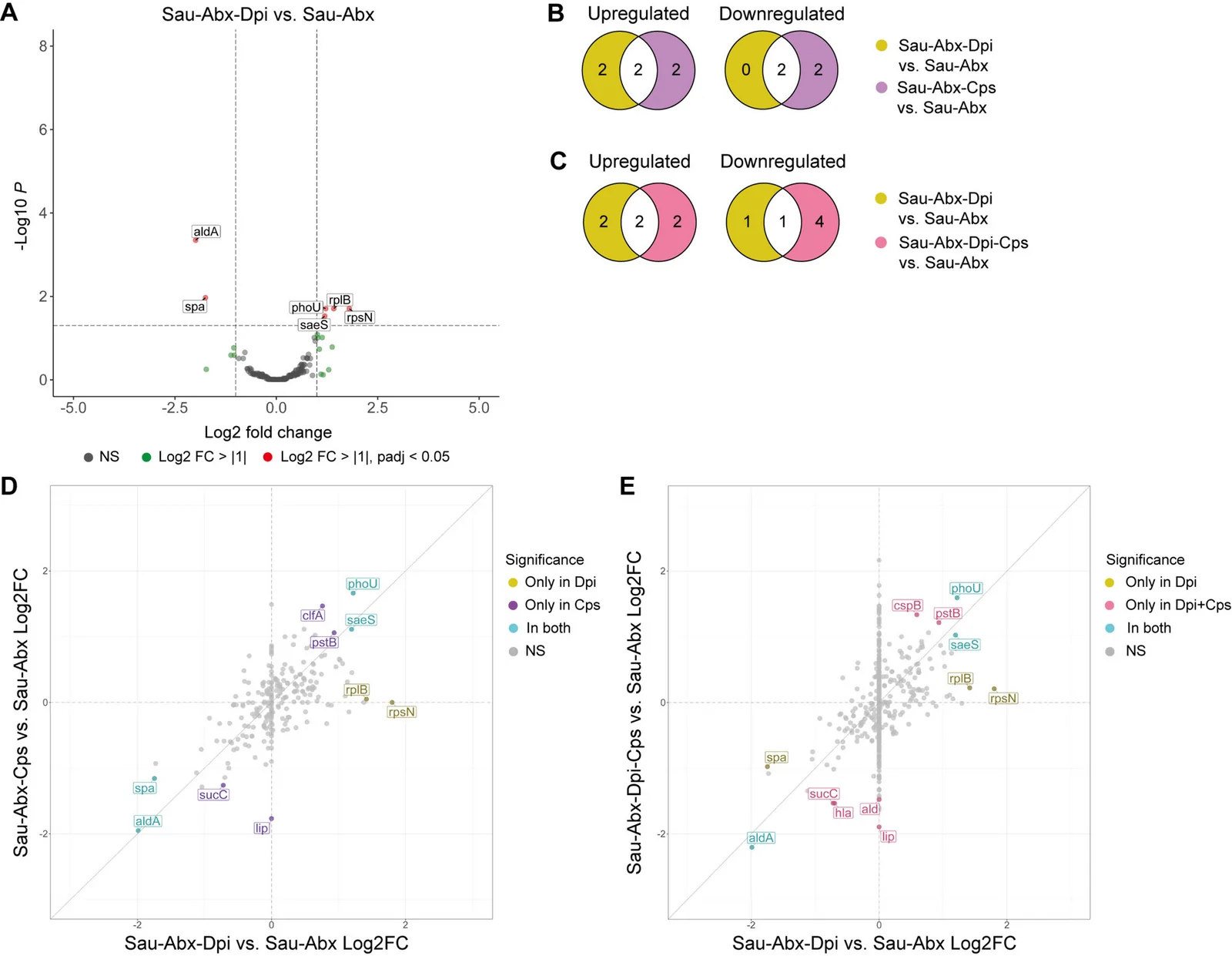
*D.* *pigrum* modulates *S*. *aureus* pathogenesis during antibiotic treatment in the airway tissue model. **A** Volcano plot showing differential expressed genes up- and down-regulated by *D. pigrum* (Dpi) as antibiotic adjuvant against *S.* *aureus* (Sau). **B**, **C** Venn diagrams summarizing *S. aureus* unique and overlapping DEGs with |FC|≥ 1. **B** Comparison of *S. aureus* unique and shared DEGs between *D.* *pigrum* and *C.* *pseudodiphtheriticum* (Cps) antibiotic adjuvant treatments against *S*.* aureus*. **C** Comparison of *S. aureus* unique and shared DEGs between *D.* *pigrum* and combined *D.* *pigrum* + *C.* *pseudodiphtheriticum* (Dpi-Cps) antibiotic adjuvant treatments against *S. aureus*. **D**, **E** Scatter plots comparing log₂ fold changes (Log2FC) in *S. aureus* transcript abundance between commensal adjuvant treatments and antibiotic treatment alone. **D** The *x*-axis represents *D.* *pigrum* and the *y*-axis represents *C.* *pseudodiphtheriticum* treatments, both in the context of *S. aureus* infection. **E** The *x*-axis represents *D*. *pigrum* and the *y*-axis represents combined *D.* *pigrum* + *C*. *pseudodiphtheriticum* treatment, both in the context of *S. aureus* infection. Blue dots indicate commonly regulated transcripts (|FC|≥ 1), whereas yellow and purple dots represent DEGs unique to commensal-specific antibiotic adjuvant treatments (*n* = 2)

Although *C. pseudodiphtheriticum* alone or in combination with *D. pigrum* KPL1914 did not enhance antibiotic efficacy against *S. aureus* in the airway tissue model, comparative dual RNA-seq profiling of these conditions provided mechanistic insight into distinct transcriptional programs associated with the unique antibiotic-potentiating effect of *D. pigrum*. To delineate these differences, we compared DEGs across the various commensal antibiotic adjuvant treatment groups (Figs. 3D–G and 4B–E). Host transcriptomic analysis revealed 22 upregulated and 15 downregulated genes uniquely associated with *D. pigrum* treatment relative to the *C. pseudodiphtheriticum* group (Fig. 3D). Specifically, *D. pigrum* selectively upregulated genes associated with scavenger receptor (e.g., *MSR1*), phagocytosis- and chemotaxis-related factors (e.g., *MYO1F*), and cell adhesion and migration molecules (e.g., *KANK4, OLFML2B*) (Fig. 3F), indicating an enhanced immune effector function. Concurrently, *D. pigrum* uniquely downregulated a pro-apoptotic factor (*BNIP*) and an ammonia transporter (*RHCG*) (Fig. 3F). Additionally, several non-coding regulatory RNAs, including *SUGCT-AS1*, *MIR493HG*, and *5_8S_rRNA*, were selectively modulated in the *D. pigrum* treatment group (Fig. 3F).

In contrast, *C. pseudodiphtheriticum* uniquely induced genes associated with immune cell trafficking (*CCR7*) and immune inhibitory receptor signaling (*CD300LF*), while attenuating the expression of lipid metabolism-related genes (e.g., *PLA2G4E*, *PLIN4*) (Fig. 3F). Collectively, these findings highlight distinct immunoregulatory programs elicited by different nasal commensals during antibiotic adjuvant treatment, which may underlie their divergent capacities to potentiate antibiotic-mediated clearance of *S. aureus*.

When compared with the *D. pigrum* KPL1914 combined with *C. pseudodiphtheriticum* treatment group, *D. pigrum* alone showed 26 uniquely upregulated and 15 uniquely downregulated genes (Fig. 3E). Consistently, *D. pigrum* treatment specifically upregulated *KANK4* and *OLFML2B*, while downregulating *5_8S_rRNA* (Fig. 3G). Moreover, host genes involved in cytokine signaling modulation (e.g., *IL1RN, GABBR2*), tissue repair and extracellular matrix remodeling (e.g., *B3GNT7, NOG, CDH6*), and innate immunity (e.g., *PGLYRP3, A2ML1*) were differentially expressed exclusively in *D. pigrum* treatment (Fig. 3G). In contrast, the combined adjuvant treatment uniquely induced immunomodulatory and inflammatory mediators (*OSM*, *FGL2*), the pattern-recognition receptor *CLEC4E*, and the antigen-presentation molecule *HLA-DQB1* (Fig. 3G)*.* Concurrently, it uniquely downregulated genes associated with tissue homeostasis (*TMEM100*), cytokine signaling regulation (*IL13RA2*), and stress-induced immune activation (*RAET1G*) (Fig. 3G). Moreover, selective downregulation of *PLA2G4E*, involved in phospholipid remodeling and bioactive lipid mediator generation, together with *PDK4*, a regulator of pyruvate entry into the TCA cycle, suggests coordinated alteration of lipid signaling and cellular metabolic programming in the combination treatment (Fig. 3G). Collectively, these findings suggest the addition of *C.* *pseudodiphtheriticum* redirects the host response toward a more immunomodulatory and metabolically altered state, potentially attenuating the antibiotic-potentiating effect of *D. pigrum* against *S. aureus*.

### *D. pigrum* antibiotic adjuvant treatment influences *S. aureus* pathogenicity in the airway tissue model

To investigate the changes of *S. aureus* transcriptome in the presence of commensal as an antibiotic adjuvant treatment, dual RNA-seq transcriptomic analysis identified 4 upregulated and 2 downregulated *S. aureus* genes in the *D. pigrum* KPL1914 treatment group compared with antibiotic treatment alone (Fig. 4A). Among the *S. aureus* differentially expressed genes (DEGs), we observed upregulation of genes associated with ribosomal function (*rplB*), phosphate homeostasis (*phoU*), and two-component signal transduction implicated in intracellular survival (*saeS*). In contrast, *S. aureus* genes involved in metabolic and redox pathways (*aldA*) and immune evasion (*spa*) were downregulated (Fig. 4A). Similarly, treatment with *C. pseudodiphtheriticum* or *D. pigrum* plus *C. pseudodiphtheriticum* led to upregulation of *phoU*, *saeS*, and another phosphate regulon gene (*pstB*), while *aldA* and the lipase gene *lip* were downregulated (Supplementary Fig. S6). In addition, *C. pseudodiphtheriticum* antibiotic adjuvant treatment specifically upregulated the fibrinogen-binding adhesin gene *clfA* and downregulated the nutrient import-associated gene *susC* (Supplementary Fig. S6A). Furthermore, *D. pigrum* and *C. pseudodiphtheriticum* combination treatment induced upregulation of the stress response gene *cspB* and downregulation of the cytolytic toxin gene *hla* (Supplementary Fig. S6B). To further identify *S. aureus* gene sets uniquely regulated by *D. pigrum*, we compared DEGs across the various commensal antibiotic adjuvant treatment groups (Supplementary Fig. 4B–E). The comparison showed that *D. pigrum* treatment uniquely led to upregulation of *S. aureus* ribosomal machinery *rplB* and *rpsN* (Fig. 4D, E), suggesting a response to translational or metabolic stress. Altogether, these findings demonstrate that the presence of commensals during antibiotic adjuvant treatment modulates *S. aureus* metabolic, redox, immune evasion, and cytotoxic capabilities, indicating a broad attenuation of bacterial virulence under commensal influence.

### Direct modulation of *S. aureus* transcription by *D. pigrum* during co-culture

**Fig. 5:**
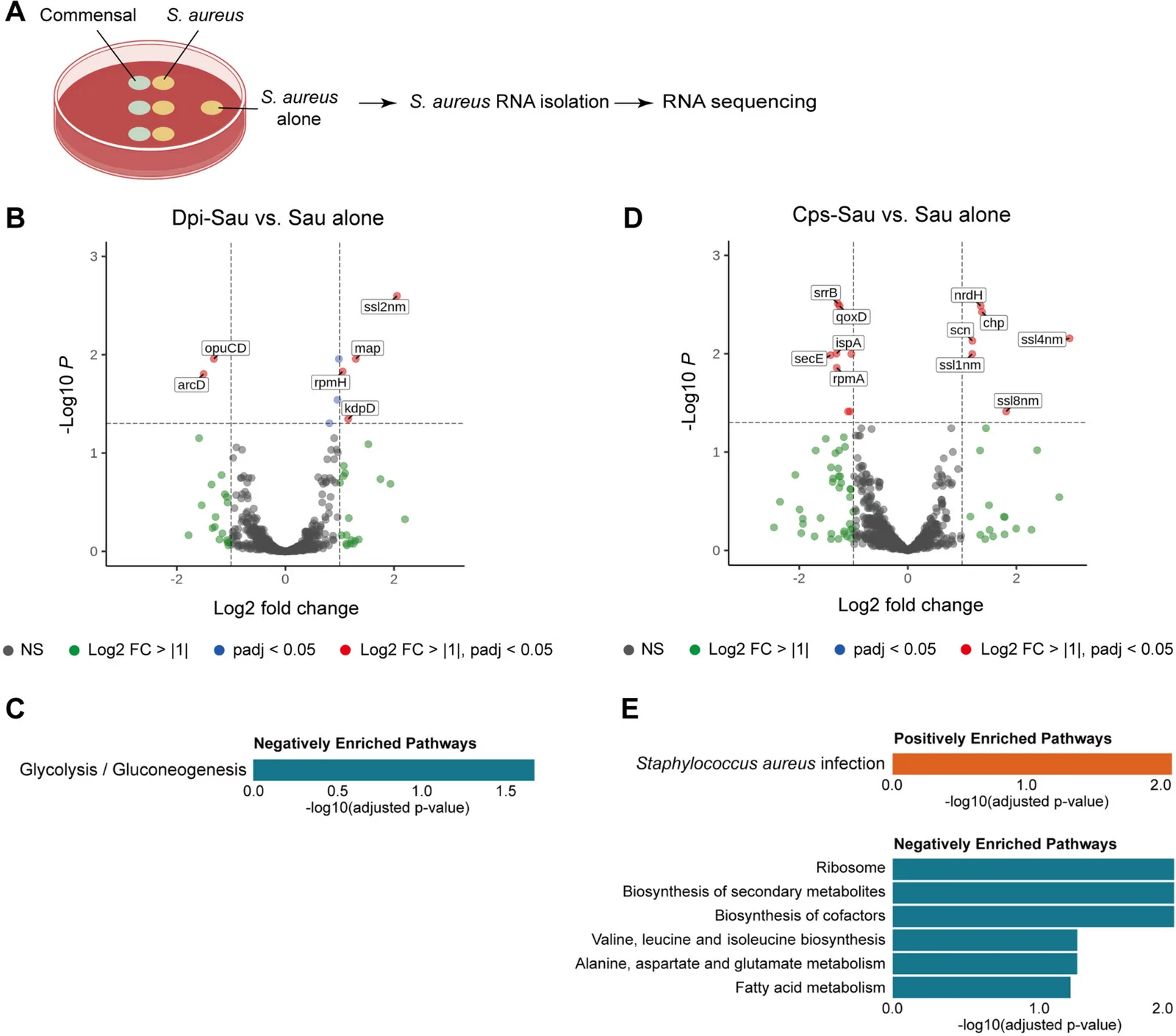
Regulation of *S*.* aureus* metabolism by *D*.* pigrum* during co-culture. **A** Schematic of experimental setup of *S. aureus* transcriptomic profiling during co-culturing with commensals on the blood agar plate. **B** Volcano plot showing *S*.* aureus* (Sau) differentially expressed genes upregulated and downregulated during co-culture with *D.* *pigrum* (Dpi). **C** Negatively enriched KEGG pathway regulated by *D*. *pigrum*. **D** Volcano plot showing *S*.* aureus* differentially expressed genes upregulated and downregulated during co-culture with *C*. *pseudodiphtheriticum* (Cps). **E** Positively and negatively enriched KEGG pathways regulated by *C*. *pseudodiphtheriticum* (*n* = 3)

Our dual RNA-seq data from the airway tissue model demonstrated that commensals influence *S. aureus* transcriptional networks. To further decipher the direct bacterial component of this interaction, we next examined *S. aureus* transcriptomics via RNA sequencing during co-culture with commensal bacteria on blood agar (Fig. 5A). In the presence of *D. pigrum* KPL1914, *S. aureus* exhibited upregulation of Staphylococcal superantigen-like protein 2 (*ssl2nm*), secreted ECM-binding protein (*map*), 50S ribosomal protein (*rmpH*), and sensor kinase of the KdpDE two-component system (*kdpD*) (Fig. 5B). These transcriptional changes suggest that *S. aureus* undergoes an adaptation characterized by enhanced host interaction and immune modulation. Concurrently, genes encoding the OpuC permease component (*opuCD*) and arginine/ornithine transporter (*arcD*) were downregulated (Fig. 5B), potentially reflecting altered nutrient availability or shifts in metabolic demands under competitive conditions during direct interaction with *D. pigrum* KPL1914. In line with this, GSEA revealed a negatively enriched KEGG pathway for glycolysis/gluconeogenesis, further supporting the presence of metabolic reprogramming in *S. aureus* during co-culture with *D. pigrum* KPL1914 (Fig. 5C).

During co-culture with *C.* *pseudodiphtheriticum* KPL1989, *S. aureus* upregulated genes involved in DNA synthesis and repair (*nrdH*) and several immune evasion factors, including the complement inhibitor (*scn*), chemotaxis inhibitory protein (*chp*), and multiple Staphylococcal superantigen-like proteins (*ssl1nm*, *ssl4nm*, *ssl8nm*), suggesting enhanced stress adaptation and virulence potential (Fig. 5D). Concurrently, genes encoding the sensor kinase of the SrrAB two-component system (*srrB*), cytochrome aa3-type quinol oxidase subunit (*qoxD*), geranylgeranyl pyrophosphate synthase (*ispA*), protein translocation subunit (*secE*), and RNA methyltransferase A (*rmpA*) were downregulated in the presence of *C. pseudodiphtheriticum*, suggesting altered energy metabolism and reduced aerobic respiratory capacity in *S. aureus* (Fig. 5D). GSEA further revealed positive enrichment of the KEGG pathway *Staphylococcus aureus* infection, along with negative enrichment of ribosomal and multiple anabolic/metabolic pathways. These findings indicate that *C.* *pseudodiphtheriticum* promotes immune evasion programs in *S. aureus* while concurrently suppressing growth-associated biosynthetic functions, consistent with a stringent-like metabolic state (Fig. 5E). Together, these results indicate that commensal species exert distinct modulatory effects on *S. aureus* transcriptional networks. *D. pigrum* primarily constrains core metabolic activity, while *C. pseudodiphtheriticum* enhances immune evasion and stress adaptation pathways, highlighting divergent ecological strategies of commensal modulation of pathobiont behavior.

## Discussion

*Staphylococcus aureus*-associated chronic infectious diseases pose a major therapeutic challenge owing to antimicrobial resistance, recurrent infection, and antibiotic-driven disruption of the resident microbiota. In this study, we demonstrate that the specific nasal commensal *D. pigrum* isolate works as a potent antibiotic adjuvant that limits *S. aureus* infection by modulating host innate immune responses and directly influencing *S. aureus* virulence and metabolic programs. By integrating epithelial reporter assays, a humanized airway tissue model, and dual RNA-seq and RNA-seq analyses, our findings provide mechanistic insight into how a commensal bacterium reshapes host-pathobiont interactions within a host-relevant environment*.*

Initial screening using the A549 Dual™ reporter assay revealed that respiratory commensals and pathobionts elicit distinct innate immune signaling activation phenotypes. *D. pigrum* induced minimal NF-κB/IRF activation and low IL-8 induction, aligning with the broader concept that commensal colonizers can occupy the mucosal niche without driving explicit inflammation [66]. Importantly, in nasal organoids, species-specific epithelial responses in cytokine and chemokine production have been shown [55], supporting the idea that nasal commensals and pathobionts differ in their colonization capacity and ability to induce cytokine responses.

Despite its limited direct activation of A549 Dual™ innate signaling, *D. pigrum* markedly enhanced antibiotic efficacy against *S. aureus* in a humanized airway tissue model. Within the tissue model, *D. pigrum* significantly reduced *S. aureus* survival when combined with flucloxacillin, preserved epithelial barrier integrity, and augmented production of pro-inflammatory cytokines such as TNF-α and IL-1β. These observations are consistent with the emerging therapeutic rationale that restoring or supplementing commensals live biotherapeutics may complement antibiotics, potentially counteracting antibiotic-associated dysbiosis and immune dysregulation discussed in CRS-focused microbiome literature [67].

The requirement for viable *D. pigrum*, as heat-killed bacteria failed to confer these benefits, underscores the importance of active bacterial metabolism, secreted effectors or metabolites, and dynamic host-bacterial or bacterial-bacterial interactions rather than passive microbial components. Notably, this effect was not reproduced by other commensals tested and was not attributable to lactic acid production, indicating that *D. pigrum* employs distinct mechanisms to potentiate host defense and antibiotic efficacy.

To further elucidate the mechanistic basis of the antibiotic-potentiating effect of *D. pigrum*, we applied dual RNA-seq to enable simultaneous, unbiased profiling of host and bacterial transcriptomes, in the airway tissue model. *D. pigrum* antibiotic adjuvant treatment enriched host inflammatory pathways, specifically increasing the expression of antigen presentation machinery and uniquely upregulating gene programs related to scavenger receptor-mediated recognition, phagocytic machinery, and chemotactic signaling. Concurrently, *D. pigrum* adjuvant treatment suppressed a pro-apoptotic molecule that could compromise immune effector functions during adjuvant treatment. These signatures suggest that *D. pigrum* promotes a tissue state characterized by enhanced recognition and clearance of bacteria.

Furthermore, *D. pigrum* uniquely regulated a subset of non-coding RNAs implicated in inflammation and immune responses. For example, *MIR493HG* encodes miR-493-5p and miR-493-3p, which have been reported to regulate Th9 cell differentiation via FOXO1 in allergic asthma [68] and CXCL10 secretion through autophagy modulation [69], respectively. These findings suggest previously unrecognized layers of non-coding RNA-mediated immune regulation that may contribute to the protective effects of *D. pigrum*. In contrast, *C. pseudodiphtheriticum* engages a distinct immunomodulatory program. This includes an upregulation of *CD300LF*, an inhibitory receptor that negatively regulates Toll-like receptor signaling via SHP-1 and SHP-2 recruitment [70–72]. This inhibitory signaling axis may attenuate the host immune activation required for effective *S. aureus* clearance, which may partly limit the capacity of *C. pseudodiphtheriticum* to enhance antibiotic efficacy.

Among the pathways enhanced by *D. pigrum*, the enrichment of IL-6-JAK-STAT3 signaling is particularly notable given its established role in mucosal immunity. In macrophages, JAK-STAT signaling drives phagosome maturation required for *S. aureus* eradication [73] and sustains pathogen-defense gene expression and bacterial killing [74]. In airway epithelium, STAT3 activated by IL-6 family cytokines is required for pulmonary host defense, its loss causing the recurrent pneumonias of STAT3 deficiency [75], and drives clearance of MRSA through antimicrobial effectors such as Reg3γ [76]. The importance of this pathway is further underscored by its active subversion by respiratory pathogens, including human metapneumovirus [77]. Accordingly, the enrichment of this hallmark program under *D. pigrum* adjuvant treatment suggests that the commensal enhances epithelial- and macrophage-associated host defense at the barrier tissue, consistent with the improved *S. aureus* clearance we observe. The enriched host programs may also relate to *S. aureus* adhesion. *S. aureus* adheres to and invades host cells largely through a fibronectin bridge linking bacterial fibronectin-binding proteins to host fibronectin and integrin α5β1 [78]. This axis is linked to interferon signaling, as IFN-γ modulates β1-integrin/fibronectin-dependent *S. aureus* binding and internalization [79], and host cells can regulate it in a defensive manner, with macrophages suppressing fibronectin expression upon infection to limit attachment [80]. Collectively, these findings suggest that the IFN-γ response and broader immune enhancement under *D. pigrum* treatment may converge on the fibronectin-integrin axis to influence *S. aureus* adhesion. However, whether the cell types in our model engage this mechanism remains to be determined.

Beyond these host-directed effects, *D. pigrum* exerted direct effects on *S. aureus* transcriptional programs both in the airway tissue model and during direct co-culture on blood agar. In the host-like environment, *S. aureus* displayed downregulation of genes involved in metabolism, redox balance, immune evasion, and cytotoxicity, indicative of attenuated virulence potential. Bacterial co-culture experiments further demonstrated that *D. pigrum* specifically constrained central metabolic pathways in *S. aureus*. These transcriptional changes are indicative of reduced metabolic efficiency and adaptive capacity, supporting the idea that *D. pigrum* can limit *S. aureus* virulence through influencing physiological fitness beyond simply inhibiting its growth. In contrast, *C. pseudodiphtheriticum* promoted *S. aureus* infection-associated pathways and regulated distinct metabolic programs, underscoring that commensal species can exert divergent effects on pathobiont behavior.

There are several limitations in this study. First, antibiotic adjuvant experiments in the tissue model were performed using a methicillin-susceptible *S. aureus* strain. Given the widespread prevalence and association of methicillin-resistant strains with CRS and skin and soft tissue infections depending on local epidemiology [3–6], it remains to be determined whether commensal-based adjuvant strategies would be equally effective against resistant strains. This is particularly important because MRSA strains may possess additional adhesion factors, immune-evasion mechanisms, toxins, and antibiotic stress-response pathways that could alter their interactions with both airway epithelium and nasal commensals [27, 81, 82]. Nevertheless, the antibiotic adjuvant effect observed here was associated with *D. pigrum*-mediated potentiation of epithelial and innate immune responses as well as metabolic suppression of *S. aureus*. These observations suggest that some aspects of the underlying mechanism may also be relevant to antibiotic-resistant *S. aureus* strains, although this remains to be tested experimentally. Supporting this possibility, recent work by Tepekule et al. [52] demonstrated that a nasal commensal-based microbiota-targeted approach can reduce the likelihood of MRSA recolonization in both computational modeling and in vitro nasal swab experiments, supporting the potential applicability of this approach to antibiotic-resistant *S. aureus* strains. Future studies should test representative MRSA lineages in the airway tissue model to determine whether *D. pigrum*-mediated immune enhancement, metabolic suppression, and antibiotic adjuvant capability are conserved across resistant *S. aureus* strains. In addition, our experiments employed a defined, reductionist community in the airway tissue model. While this enabled us to isolate the host responses attributable to individual commensals and to a defined two-member combination, the human nasal microbiota is considerably more complex and includes fungal, viral, and additional bacterial members whose composition varies between hosts. How these commensal-potentiated adjuvant effects occur within the intact ecosystem therefore remains to be established. Second, the antibiotic used here, flucloxacillin, is a first-line anti-staphylococcal penicillin in several regions but is not approved in all jurisdictions (e.g., the U.S. and Canada). Confirming the commensal adjuvant effect with other β-lactam and non–β-lactam anti-staphylococcal agents would further support its clinical generalizability. Third, while transcriptomic signatures strongly suggest reduced metabolic and pathogenic fitness of *S. aureus*, direct measurements of growth dynamics, nutrient competition, and metabolic flux would further strengthen these findings. Additionally, although the airway tissue model captures key features of human immunity, in vivo studies will be necessary to assess colonization dynamics, long-term safety, and therapeutic efficacy in complex host environments. In particular, the durability of the commensal adjuvant effect was not assessed in the present study. Testing the sustainability of this effect, and its relationship to protection against *S. aureus*-associated remote infections such as skin and soft tissue infections, will be an important future direction.

Collectively, our data support a dual-mechanistic model in which *D. pigrum* KPL1914 constrains *S. aureus* through (i) indirect enhancement of host immune responses and (ii) direct suppression of bacterial pathogenic and metabolic programs. This multifaceted mode of action distinguishes *D. pigrum* from traditional antimicrobial strategies and highlights the therapeutic potential of manipulating commensal-pathobiont interactions to overcome antibiotic resistance. Together, these findings advance our understanding of commensal-mediated control of pathobionts and support the development of microbiota-based adjuvant therapies for chronic airway infections.

## Methods

### Bacterial isolates and culture

All the bacterial isolates used in this study are listed in Table 1. Species identification was done with MALDI-TOF biotyping.

To obtain the exponentially grown bacteria for reporter assays and 3D tissue model experiment, commensal *D. pigrum*, *Corynebacterium* spp., and *C. avidum* were harvested from 48 h-incubated Columbia agar + 5% sheep blood (BIOMERIEUX #43,049) at 37 ℃ with 5% CO_2_ and inoculated in brain heart infusion (BHI) medium at OD600_nm_ 0.1 in a stagnant 37 ℃ environment for 6 h before being utilized for the stimulation experiment. Overnight tryptic soy broth (TSB) liquid culture of *S. epidermidis*, *S. aureus*, and *M. catarrhalis* were diluted 1:10 in the fresh TSB and cultured at 37 ℃ with 220 rpm for 2 h before being utilized for the stimulation experiment. *S. pneumoniae* were harvested from 12 h-incubated Columbia agar + 5% sheep blood and inoculated in Todd Hewitt broth + 2% yeast extract at OD600_nm_ 0.1 in a stagnant 37 ℃ environment for 4 h before being utilized for stimulation experiment.

### A549 NF-κB and IRFs reporter assays

The A549 Dual^TM^ reporter cell line was purchased from InvivoGen (a549d-nfis) and handled according to the manufacturer instructions. Briefly, the A549 Dual^TM^ cells were maintained in the growth medium (DMEM, 2 mM L-glutamine, 4.5 g/l glucose, 10% fetal bovine serum (FBS), 100 U/ml–100 μg/ml Pen-Strep, 100 μg/ml Normocin™) with antibiotics Blasticidin and Zeocin® to select cells with integrated inducible reporter constructs.

Before the bacterial stimulating experiment, the cells were cultured in the assay medium (DMEM, 2 mM L-glutamine, 4.5 g/l glucose, 10% heat-inactivated FBS). For the A549 reporter assays, the exponentially grown bacteria were harvested and adjusted to the multiplicity of infection (MOI) 1 for stimulation. To measure NF-κB induction, the supernatant was collected after 18–24 h bacterial stimulation and transferred to a fresh flat-bottom 96-well plate, followed by the addition of QUANTI-Blue™ to determine the NF-κB-induced secreted alkaline phosphatase (SEAP) levels by microplate reader at 655 nm (SpectraMax iD3). To measure IRF induction, the supernatant was collected after 18–24 h bacterial stimulation and transferred to a fresh 96-well black plate. QUANTI-Luc™ 4 Reagent was used to determine the IRF-induced activity of Lucia luciferase by the microplate reader (SpectraMax iD3). One nanogram per milliliter of TNF-α and IFN-α were used as positive controls for NF-κB and IRF induction, respectively. In *S. aureus* and *S. pneumoniae* stimulation experiments, 4X MIC of flucloxacillin was added 3 h post-stimulation to prevent bacteria from damaging the host cells.

### ELISA

The levels of cytokines IL-8, IL-1β, and TNF-α were determined according to manufacturer instructions using human IL-8/CXCL8 DuoSet, human IL-1 beta/IL-1F2 DuoSet, human TNF-alpha DuoSet ELISA kit (R&D Systems) respectively. Supernatants collected from A549 reporter assay were used undiluted to determine IL-8 levels. Supernatants collected from 3D airway tissue model were diluted 1:500 with diluent reagent to determine IL-8 levels, and undiluted supernatants were used to determine IL-1β, and TNF-α levels in the airway tissue model. All measurements were performed in technical duplicates and concentration was calculated and analyzed based on the standard curved.

### Measurement of LDH release

The supernatants collected from the A549 reporter assay were used undiluted to determine LDH release by using CytoTox 96® Non-Radioactive Cytotoxicity Assay (Promega) and measured by SpectraMax iD3 at 490 nm according to the manufacturer instructions.

### Establishment of humanized airway tissue model

The airway tissue model was established as previously described [63–65]. Briefly, lung fibroblast cells MRC-5 were gently mixed with collagen I (Advanced BioMatrix) to form the basis of the stromal matrix in the transwell (Corning #353,092) in 6-well plate (Corning #353,046). After 7 days of incubation at 37 ℃ with 5% CO_2_ humidified atmosphere, human monocytes were added to the surface of stromal matrix, followed by the overnight incubation. Human bronchial epithelial 16HBE cells were then added to the stromal matrix and incubated for 4 days. Finally, the model was air-exposed by removing the cell culture medium in the transwell and incubated for an additional 7 days to obtain a mature humanized airway tissue model. The culture medium (DMEM, 2 mM L-glutamine, 1× non-essential amino acid (Thermo Fisher Scientific), 1 M HEPES (Thermo Fisher Scientific), 10% heat-inactivated FBS, and 100 U/ml–100 μg/ml Pen-Strep) was refreshed every 2 days throughout the entire setup of tissue model. The antibiotic-free media were used during the last refreshment.

### Commensal antibiotic adjuvant treatment, tissue decomposition, bacterial harvest, and colony-forming unit enumeration

Antibiotic treatment was performed by replacing the antibiotic-free medium in the lower chambers of the air–liquid interface system with an antibiotic-containing medium (4× MIC of flucloxacillin) for 3 h after *S. aureus* Cowan I infection for 6 h, followed by the addition of commensal bacteria to the upper chambers of the airway tissue model for 16 h. Across all adjuvant conditions, *S. aureus* was inoculated at a fixed burden (1 × 10^6^ CFU) and the total commensal load was held constant at 5 × 10^7^ CFU (single-commensal treatments) or 2.5 × 10^7^ CFU each of *D.* *pigrum* and *C. pseudodiphtheriticum* (combination), such that the commensal-to-pathogen ratio and total bacterial burden were equivalent across treatments. Following commensal antibiotic adjuvant treatment, the airway tissue was chopped and digested in RPMI 1640 containing 2 mg/ml collagenase D and 50 U/ml of DNase I. For assessing bacterial survival, the chopped tissue piece was incubated in the digestion buffer at 37 ℃ for 30 min with shaking at 1400 rpm. The digested tissue was centrifuged at 14,000 rpm for 5 min, followed by PBS wash for two times. The cell pellet was suspended in PBS, serial diluted, and drop-platted on the blood agar plate for 24 h at 37 ℃.

### Histology

Tissue samples were fixed in 4% paraformaldehyde, dehydrated and embedded in paraffin. Paraffin blocks were then sectioned at 4 µm thickness using a microtome for histological and immunohistochemical analysis. Hematoxylin and eosin (H&E) staining was performed to evaluate cellular and tissue morphology, while Brown-Brenn (BrB) modified Gram staining was used for bacterial identification. H&E staining was carried out using the Tissue-Tek® Prisma™ automated system. BrB staining was performed manually with the following reagents: 1% crystal violet (Artechemis), 5% sodium bicarbonate (Artechemis, stored at 4 °C), saturated aqueous basic fuchsin (1%) (Artechemis), Lugol's iodine solution (Artechemis), 0.1% picric acid in acetone (Morphisto), and acetone (Artechemis, Thommen-Furler AG).

### Determination of L-lactate

The L-lactate determination in the tissue model supernatant was following the manufacturer’s instructions (Cat. No.11112821035, Boehringer Mannheim, R-biopharm).

### Commensal-pathobiont co-culture assays

For co-culture conditions, 10 µL of each commensal bacterial suspension (*D. pigrum* KPL1914 and *C. pseudodiphtheriticum* KPL1989; OD₆₀₀ = 0.3) were spotted adjacent to 5 µL of the *S. aureus* (Newman) suspension (OD₆₀₀ = 0.05) on a 0.2 µm pore-size, 47 mm-diameter polycarbonate membrane placed on a COS agar plate. Multiple replicate spots were used for each condition. Polycarbonate membranes were employed to permit nutrient exchange between bacterial cells and the underlying agar while facilitating bacterial recovery at later time points. Co-cultures were incubated at 37 °C with 5% CO₂ for 24 h, after which bacteria were harvested from individual spots. Four spots per condition were pooled and stored overnight at 4 °C in 500 µL RNAlater™ solution (Thermo Fisher Scientific). Total RNA was extracted using the RNeasy Mini Kit (Qiagen, Germany), followed by DNA removal with the TURBO DNA-free Kit (Invitrogen, MA, USA). RNA quantity and quality were assessed using the Agilent TapeStation System (Agilent, MA, USA). The experiment was performed in biological triplicate. RNA samples were processed by Vertis Biotechnologie AG (Freising, Germany) for rRNA depletion, RNA fragmentation, strand-specific cDNA library preparation, and Illumina NextSeq 500 single-end sequencing (> 2 million reads per sample).

### Dual RNA sequencing and data analysis

3D humanized airway tissue models were sectioned into small pieces, submerged in approximately five volumes of RNAlater™ solution (Thermo Fisher Scientific), and stored at − 80 °C until further processing. Dual RNA sequencing was performed by Eurofins Genomics (Germany). Briefly, total RNA was extracted from entire tissue pieces, followed by simultaneous depletion of host and bacterial ribosomal RNA and preparation of strand-specific cDNA libraries. RNA sequencing of airway tissue models was conducted using 150-bp paired-end reads on an Illumina NovaSeq platform with the RNA_HMR_BacDepl library preparation protocol.

RNA-seq analysis pipeline was implemented utilizing Snakemake (v7.32.4) [83] for both bacterial and human transcriptomes. Raw sequencing data underwent quality assessment with FastQC (v0.12.1) [84] and preprocessing using fastp (v0.23.2) [85, 86] for adapter removal and quality filtering. Quality-filtered reads from tissue models were aligned to the human reference genome (GRCh38) using splice-aware HISAT2 (v2.2.1) [87], with unmapped reads subsequently aligned to bacterial reference genomes using adjusted parameters. Blood agar sample reads were directly mapped to bacterial reference genomes using bowtie2 (v2.5.2) [88]. Gene expression quantification was performed with featureCounts (v2.0.8) [89] using appropriate genomes for both human and bacterial samples. The pipeline’s integrity was validated using MultiQC (v1.17) [90]. Differential gene expression analysis was conducted in R (v4.4.3) using DESeq2 (v1.46.0) [91] with a multifactor design to account for donor effects in tissue models. Genes with absolute log2 fold change > 1 and adjusted *p*-value < 0.05 were classified as significantly differentially expressed. Visualization of results included volcano plots generated using EnhancedVolcano (v1.24.0) [92], while gene set enrichment analysis was performed with clusterProfiler (v4.14.6) [93] for both KEGG [94] and Hallmark [95] gene set collections to identify significantly enriched biological pathways. Additional visualizations including heatmaps and scatterplots were generated using ggplot2 (v3.5.2) [96].

### Statistical analysis

Data were analyzed using GraphPad Prism software v.10 (GraphPad Software Inc, San Diego, CA, USA) and the statistical methods used are indicated in the respective figure legend.

## Supplementary Information

Supplementary Material 1: Figure S1. Activation of innate signal transduction and LDH release by nasal commensals and pathobionts in A549 Dual™ cells. (A–D) A549 Dual™ cells were stimulated with commensals or pathobionts at MOI 1 for 18–20 h. Induction of NF-κB and IRF were quantified by alkaline phosphatase (SEAP) and Lucia luciferase activity in the supernatant, respectively. Release of IL-8 and LDH were measured by ELISA and cytotoxicity assay in the supernatant, respectively. *S. aureus* and *S. pneumoniae* stimulating wells were added with 4X MIC of flucloxacillin 3 h post-stimulation. (Data represent mean ± SEM. *n* = *2–6*). Figure S2. Survival of nasal commensals or pathobionts and L-lactate production in the airway tissue model. (A) The airway tissue model was colonized with commensals or pathobionts for 18 h. Percentage of bacterial survival was calculated by normalization to inoculum. (Data represent mean ± SEM.*n* = *3–4*) (B) L-lactate was quantified in the supernatant of the airway tissue model collected after 16 h with or without addition of commensal bacteria. (Data represent mean ± SEM. *n* = *3–9*). *P* values are indicated (unpaired t-tests without correction for multiple testing). Figure S3. Barrier integrity of the airway tissue model during commensal adjuvant therapy. (A–B) Three representative hematoxylin and eosin (H&E)-stained (A) and Brown-Benn (BrB)-stained (B) histological sections of the airway tissue model following treatment with *D. pigrum* (Dpi), *C.* *pseudodiphtheriticum* (Cps), or the combined treatment (Dpi + Cps) during *S. aureus* (Sau) infection. Antibiotic treatment alone (Abx) served as the control. (C-D) Three representative H&E-stained (C) and BrB-stained (D) histological sections of the airway tissue model that were unstimulated (Unstim), infected with *S. aureus* (Sau), or colonized with *D.* *pigrum* (Dpi), or *C.* *pseudodiphtheriticum* (Cps). Figure S4. Enriched hallmark pathways regulated by *D. pigrum *as antibiotic adjuvant. Heatmap showing genes involved in enriched hallmark pathways. Values represent Log_2_-fold change. Figure S5. Dual RNA-seq profiling host transcriptomics in the presence of commensals as antibiotic adjuvant treatment against *S. aureus *in the airway tissue model. (A) Volcano plot showing differentially expressed genes up- and down-regulated by *C.* *pseudodiphtheriticum* (Cps) as antibiotic adjuvant against *S. aureus* (Sau). Top 10 upregulated and top 5 downregulated genes are shown. (B) Enriched hallmark pathways regulated by *C.* *pseudodiphtheriticum* as antibiotic adjuvant against *S. aureus*. (C) Volcano plot showing differentially expressed genes up- and down-regulated by *D.* *pigrum* + *C.* *pseudodiphtheriticum* (Dpi-Cps) as antibiotic adjuvant against *S. aureus*. Top 10 up-regulated and top 5 down-regulated genes are shown. (D) Enriched hallmark pathways regulated by *D.* *pigrum* + *C.* *pseudodiphtheriticum* as antibiotic adjuvant against *S. aureus*. (*n* = *2*). Figure S6. Dual RNA-seq profiling *S. aureus* transcriptomics in the presence of commensals as antibiotic adjuvant treatment in the airway tissue model. (A) Volcano plot showing differentially expressed genes up- and down-regulated by *C. pseudodiphtheriticum* (Cps) as antibiotic adjuvant against *S. aureus* (Sau). (B) Volcano plot showing differential expressed genes up- and down-regulated by *D. pigrum* + *C. pseudodiphtheriticum* (Dpi-Cps) as antibiotic adjuvant against *S. aureus*. (*n* = *2*).

## Data Availability

Data supporting the findings of this study are available in the European Nucleotide Archive under accession number PRJEB107788. All code used for all analyses is openly available at GitHub (https://github.com/sdb-lab-org/Adjuvant-Commensal-Therapy).
